# Phosphorylation of Connexin36 near the C-terminus switches binding affinities for PDZ-domain and 14–3–3 proteins in vitro

**DOI:** 10.1038/s41598-020-75375-0

**Published:** 2020-10-27

**Authors:** Stephan Tetenborg, Helen Y. Wang, Lena Nemitz, Anne Depping, Alexsandra B. Espejo, Jaya Aseervatham, Mark T. Bedford, Ulrike Janssen-Bienhold, John O’Brien, Karin Dedek

**Affiliations:** 1 Animal Navigation/Neurosensorics, Institute for Biology and Environmental Sciences, University of Oldenburg, Oldenburg, Germany; 2 Ruiz Department of Ophthalmology & Visual Science, The University of Texas Health Science Center at Houston, Houston, TX 77030 USA; 3 Visual Neuroscience, Dept. of Neuroscience, University of Oldenburg, Oldenburg, Germany; 4 Department of Epigenetics and Molecular Carcinogenesis, University of Texas M.D. Anderson Cancer Center, Smithville, TX 78957 USA; 5 Research Center Neurosensory Science, University of Oldenburg, Oldenburg, Germany

**Keywords:** Phosphorylation, Gap junctions

## Abstract

Connexin36 (Cx36) is the most abundant connexin in central nervous system neurons. It forms gap junction channels that act as electrical synapses. Similar to chemical synapses, Cx36-containing gap junctions undergo activity-dependent plasticity and complex regulation. Cx36 gap junctions represent multimolecular complexes and contain cytoskeletal, regulatory and scaffolding proteins, which regulate channel conductance, assembly and turnover. The amino acid sequence of mammalian Cx36 harbors a phosphorylation site for the Ca^2+^/calmodulin-dependent kinase II at serine 315. This regulatory site is homologous to the serine 298 in perch Cx35 and in close vicinity to a PDZ binding domain at the very C-terminal end of the protein. We hypothesized that this phosphorylation site may serve as a molecular switch, influencing the affinity of the PDZ binding domain for its binding partners. Protein microarray and pulldown experiments revealed that this is indeed the case: phosphorylation of serine 298 decreased the binding affinity for MUPP1, a known scaffolding partner of connexin36, and increased the binding affinity for two different 14–3–3 proteins. Although we did not find the same effect in cell culture experiments, our data suggest that phosphorylation of serine 315/298 may serve to recruit different proteins to connexin36/35-containing gap junctions in an activity-dependent manner.

## Introduction

In the central nervous system, electrical synapses directly connect the cytoplasm of neighboring neurons and provide a means for fast signal transmission. Electrical synapses are formed by gap junctions, which are assembled from connexin molecules. Among these proteins, Connexin36 (Cx36) is the most abundant connexin isoform in neurons of the mammalian central nervous system. It is strongly expressed in the olfactory bulb^1^, inferior olive^2^, hippocampus^3^, and the retina^3^ and is important for signal synchronization^2,4^, network oscillation^4^, and signal-to-noise amplification^5^. Moreover, Cx36 is essential for the primary and secondary rod pathways in the retina^6–8^.

Recent evidence shows that gap junction channels are not simple intercellular channels but undergo complex, activity-dependent regulation^9–12^. In the mammalian retina, for example, coupling in AII amacrine cells is increased by calcium influx through extra-synaptic NMDA receptors, subsequent activation of Ca^2+^/calmodulin-dependent kinase II (CaMKII, presumably CaMKII-δ^13^), and phosphorylation of Cx36^9^.

**Figure 1:**
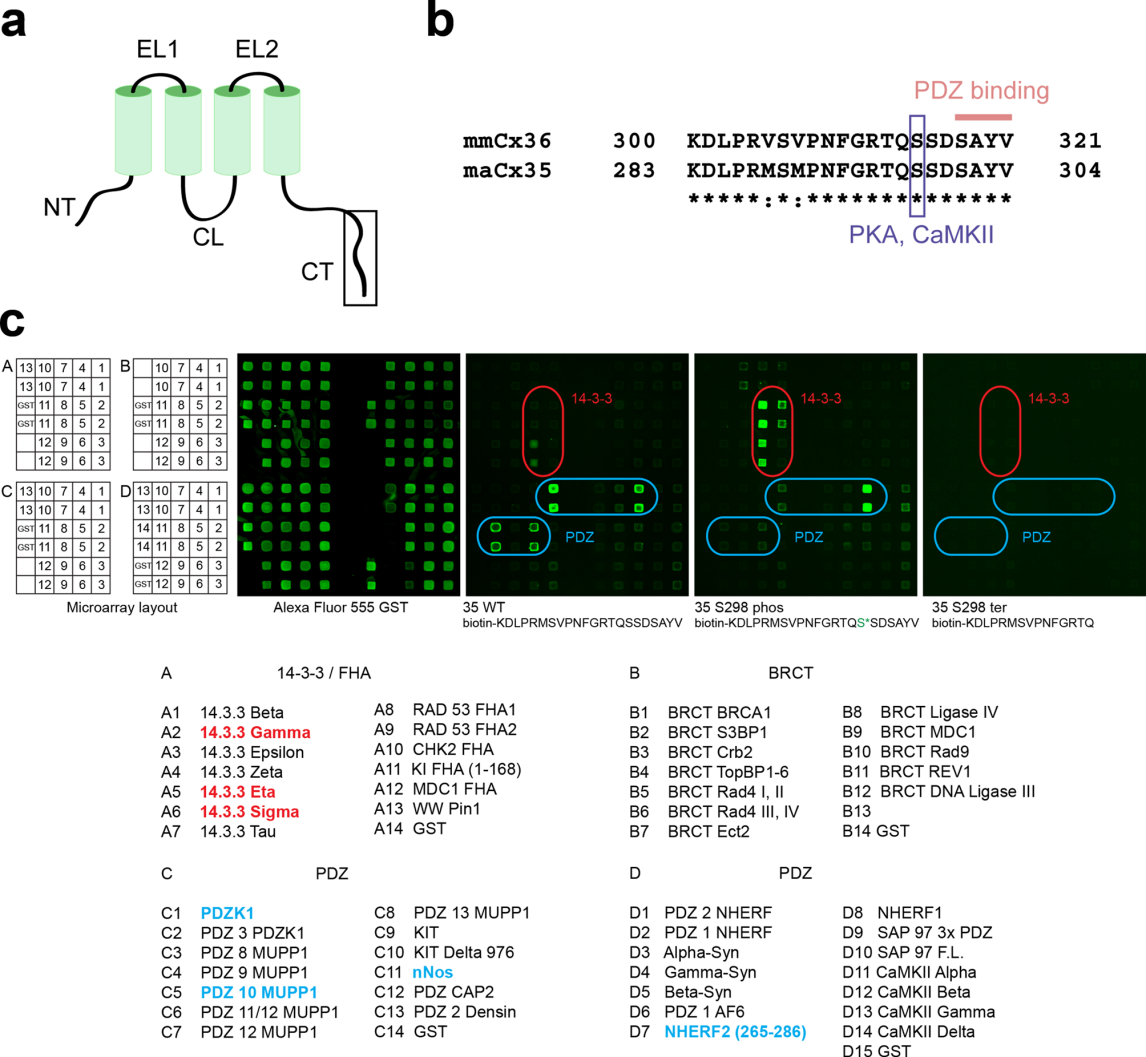
Protein microarray data indicate that phosphorylation of S298 in maCx35 may act as a functional switch. (**a**) Membrane topology of connexins. N- and C-terminal tails (NT and CT, respectively) and the cytoplasmic loop (CL) reside inside the cytoplasm. The two extracellular loops (EL) are also indicated. (**b**) Sequence of the C-terminal end of maCx35/mmCx36, as indicated by the box in (**a**). Sequence identity (*) is high for mmCx36 and maCx35 at the C-terminal end. The blue box indicates a PKA/CaMKII consensus site at position S315 and S298 for mmCx36 and maCx35, respectively. The last four amino acids represent a PDZ binding domain, present in both proteins. (**c**) Microarray layout (A-D); the respective baits (all fused to GST) are found below. An array probed with Alexa Fluor 555 anti-GST antibody served as positive control. Peptides fused to biotin containing the C-terminal tail of maCx35 (35 WT), phosphorylated maCx35 (35 S298 phos), and a truncated version (35 S298 ter) were used as probes and showed differential results, marked by rounded squares. 14–3–3 proteins (red) and NHERF2 aa265-288 (blue D7) showed an increased binding to the 35 S298 phospho probe compared to 35 WT. In contrast, PDZK1, PDZ10 of MUPP1, and nNOS (blue) decreased their binding to the 35 S298 phospho probe. The truncated 35 S298 ter probe showed no binding interactions.

Similar to chemical synapses, gap junctions form microcompartments that assemble a plethora of proteins, which regulate synaptic strength in response to pH, voltage or Ca^2+^ changes. Among these proteins, several kinases were shown to directly or indirectly modulate Cx36-containing gap junctions, e.g., protein kinase A^14–16^ (PKA) and CaMKII^9,11,17^, with different CaMKII isoforms presumably regulating Cx36 in different neuronal cell types^13^. Accordingly, the protein sequence of mouse (*Mus musculus, mm*) Cx36 contains several consensus motifs for PKA, CaMKII and other kinases^11,18^. Serine 315 (S315) constitutes a CaMKII phosphorylation site^11^, which is located close to the C-terminal end of the protein and thereby in immediate vicinity of the PDZ binding domain formed by the last four amino acids (Fig. 1a,b). This PDZ binding domain seems to be important for channel assembly^19^ and may help to recruit scaffolding proteins^20^. Both, the phosphorylation site and the PDZ binding domain are conserved among species, e.g., S298 in perch (*Morone americana*, *ma*) Cx35 (Fig. 1b), which points towards an essential function for these motifs in gap junction regulation.

Because of the close proximity of S315/298 to the PDZ binding domain at the C-terminal end of mmCx36/maCx35, respectively, we hypothesized that phosphorylation of S315/298 may regulate the binding of interaction partners to the PDZ binding domain. Two potential regulatory actions are conceivable: phosphorylation may either increase or decrease binding, providing a means to regulate the interaction with different partners in an activity-dependent way. Here, we provide evidence that such a phosphorylation-mediated switch of Cx36/35 binding partners occurs in vitro. A protein microarray was used to identify candidate proteins whose interaction may change by phosphorylation of the serine residue. Most of these candidates were tested subsequently in pull-down assays. We found that phosphorylation of S298 in maCx35 increased the interaction with 14–3–3 γ and η whereas it decreased the interaction with PDZ10 of MUPP1. Although cell culture experiments did not reveal the same effect, our data indicate that in neuronal Cx36/Cx35, phosphorylation of S315/298 may serve as a switch to recruit different scaffolding partners to the gap junction plaque.

## Results

### Protein domain microarray probed with peptides of the maCx35 C-terminal

Phosphorylation and dephosphorylation of proteins occur at serine, threonine and tyrosine residues. Both posttranslational modifications may change the protein conformation, leading to changes in protein properties, such as stability, localization and interaction with other proteins^21^. Here, we hypothesized that phosphorylation of residue S315/298 in mmCx36/maCx35 may alter the binding of proteins to the PDZ binding domain at the very C-terminal end of the protein.

To investigate this, we first performed a protein microarray study^22^. Proteins either containing PDZ domains or involved in phosphorylation detection were immobilized as GST fusion proteins on the array substrate and used as baits (Fig. 1c). The arrays were probed with three different maCx35 constructs for preys, fused to biotin and pre-conjugated to Cy3-streptavidin: (1) the wild-type C-terminus of maCx35, (2) the same sequence but with a phosphorylation at S298 (phospho-S298), and (3) the C-terminal tail truncated at S298 (S298 ter). We found that three (γ, η and σ) out of seven 14–3–3 protein isoforms and the PDZ domain of NHERF2 (aa 265–288) strongly increased their binding to the C-terminal tail of maCx35 when S298 was phosphorylated. Conversely, other PDZ domains and PDZ-containing proteins (PDZK1, PDZ10 of MUPP1, and nNOS) decreased their binding to the C-terminal tail of the phosphorylated maCx35. Control experiments with the truncated C-terminal tail of maCx35 confirmed the specificity of the interaction: none of the proteins bound to the truncated tail, in which the phosphorylation site and PDZ-binding domain were missing (Fig. 1c). Repeat experiments using mmCx36 C-terminal peptides with larger PDZ domain arrays corroborated NHERF2 results and similarly revealed phosphorylation-dependent decrease in binding to PDZ domains from Chapsyn110, PSD95 and SAP102 (Supplementary Fig. 1).

### Pulldown experiments with purified proteins

**Figure 2:**
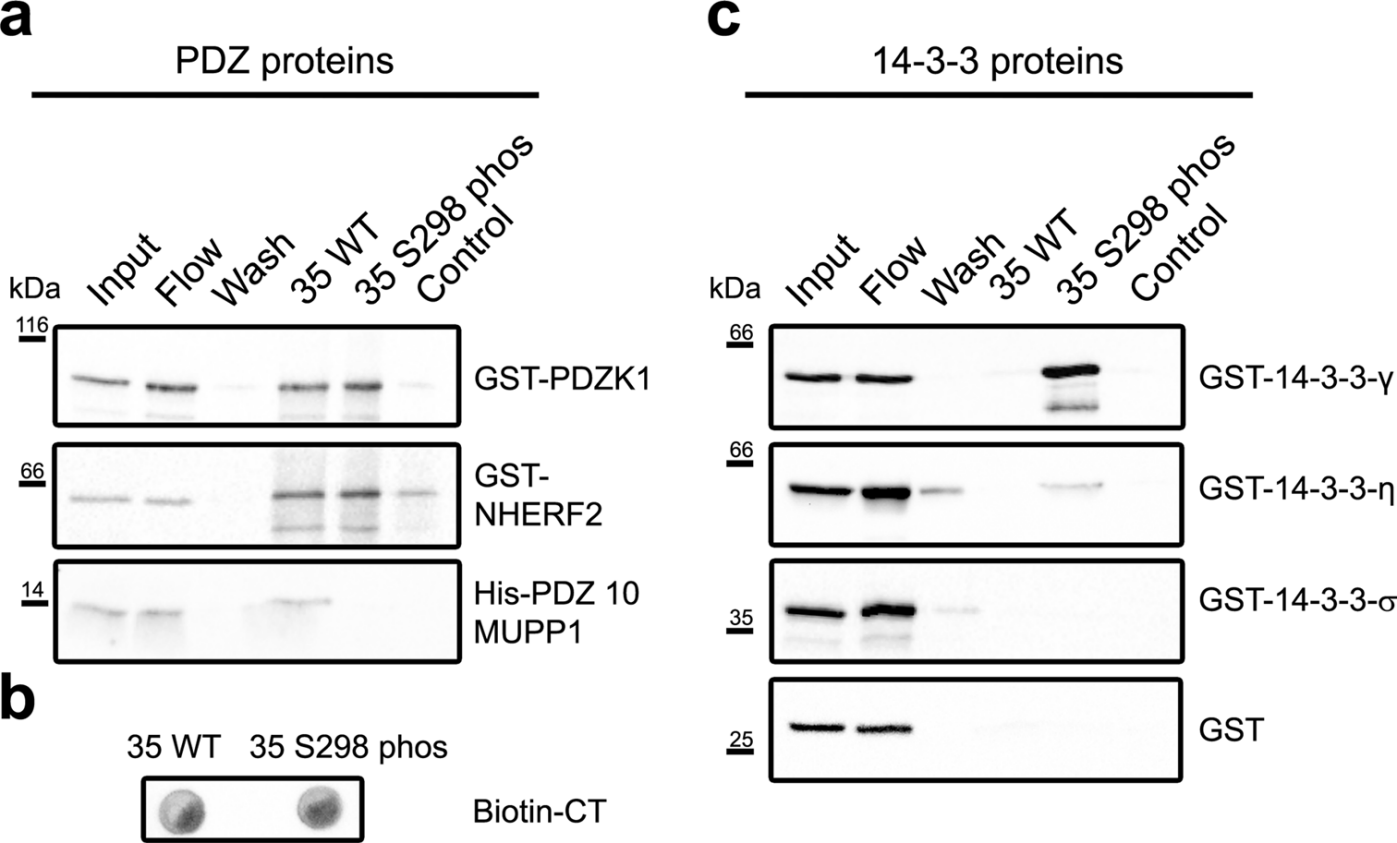
Pull-down experiments confirmed the phosphorylation-induced change in interaction of PDZ10 of MUPP1 and 14–3–3 proteins with maCx35. (**a**) Pull-down experiments using GST-coupled PDZK1, NHERF2 and the tenth PDZ domain of MUPP1 (PDZ 10 MUPP1). PDZK1 and NHERF2 bound to both WT and S298 phospho peptides, but both proteins showed residual binding to the empty beads (control). PDZ-10 MUPP1 showed decreased binding to the C-terminal tail of maCx35 when S315 was phosphorylated (35 S298 phos) compared to the non-phosphorylated form (35 WT). (**b**) Drop blot, demonstrating that phosphorylated and non-phosphorylated maCx35 constructs had similar concentrations. (**c**) GST-bound 14–3–3 proteins γ and η showed increased binding to the phosphorylated form of maCx35. 14–3–3 σ, in contrast, did not bind to the C-terminal tail of maCx35. GST alone did not show any binding to the beads. Each pull-down was performed 2–4 times.

Several of the hits in the microarray screen were then tested for interaction in pull-down experiments (see Table 1 for details on constructs). maCx35 peptides (previously used in the microarray) were coupled to streptavidin beads and incubated with individual proteins that were identified in the array. Again, we used the wild-type C-terminus and a modified version (maCx35 S298 phos) to test for phosphorylation-dependent interactions (Fig. 2; Supplementary Figs. 2 and 3). Empty beads (without conjugated peptide) served as control. We tested GST-fused PDZK1, NHERF2 and PDZ 10 of MUPP1 for interaction with the wild-type and the phosphorylated C-terminal tail of maCx35 (Fig. 2a). A drop blot was used to control for equal amounts of both maCx35 peptides (Fig. 2b). We found—consistent with the protein microarray data—that PDZ10 of MUPP1 bound strongly to the wild-type C-terminal tail of maCx35 but not to the phosphorylated form. In contrast, PDZK1 did not show differential binding to the two maCx35 constructs. NHERF2 showed a similar interaction with wild-type and phosphorylated maCx35 as PDZK1 but even bound to the empty beads so that we could not draw any conclusions about the specificity of its binding. However, our data confirmed the phosphorylation-dependent interaction of maCx35 and PDZ 10 of MUPP1.

**Table 1:** List of constructs tested in peptide pulldowns.

Construct	Description	Species
His PDZ-10 MUPP1	Tagged PDZ 10 domain of MUPP1	Mouse
GST-PDZK1	Tagged full length PDZK1	Mouse
GST-NHERF2	Tagged full length NHERF2	Mouse
GST-14–3–3-γ	Tagged full length GST-14–3–3-γ	Rat
GST-14–3–3-η	Tagged full length GST-14–3–3-η	Human
GST-14–3–3-σ	Tagged full length GST-14–3–3-σ	Human

Data from the protein microarray also suggested that 14–3–3 proteins may increase their binding to maCx35 when S298 is phosphorylated. This was confirmed by the pulldown experiments (Fig. 2c). 14–3–3 γ, η and σ (full-length) were fused to GST and tested in the same way as the PDZ domain-containing constructs. While GST alone and 14–3–3 σ did not show any binding to the maCx35 constructs or the empty control beads, γ and η strongly bound to maCx35 when S298 was phosphorylated.

Thus, our data provide evidence that MUPP1 (via PDZ 10) and 14–3–3 proteins γ and η bind differentially to the C-terminal tail of maCx35: MUPP1 increases its binding when S298 *is not* phosphorylated whereas the two 14–3–3 proteins bind stronger when S298 *is* phosphorylated. As mentioned above, this may provide a means to differentially regulate the recruitment of proteins to the gap junction site in a phosphorylation- (i.e., activity-) dependent manner.

### Does phosphorylation of S315 in mmCx36 change the size of the gap junction?

**Figure 3:**
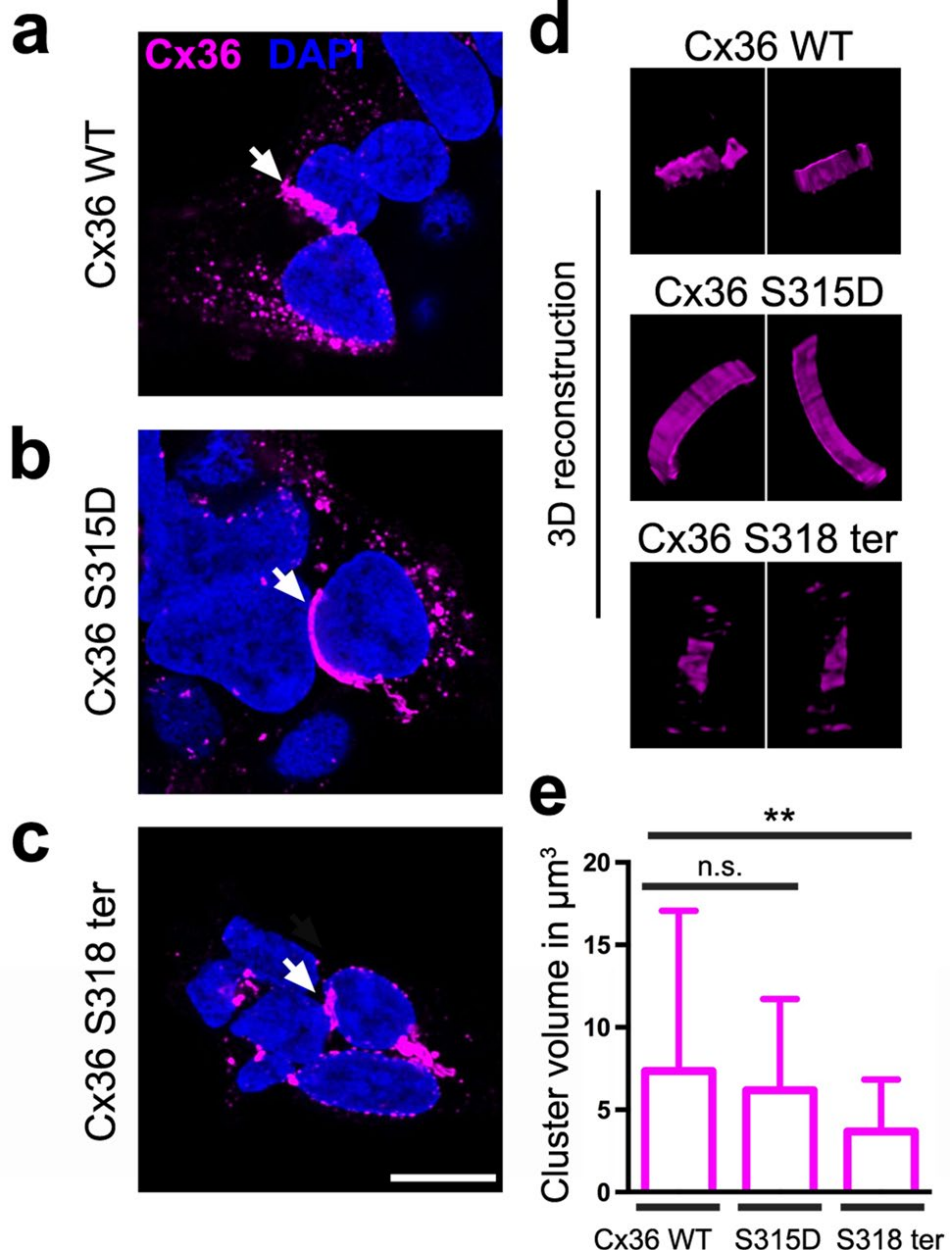
A phosphomimetic S315D mutation in mmCx36 did not change the size of mmCx36-containing gap junctions. HEK293 cells transfected either with mmCx36 WT (**a**), the S315D mutant (**b**), which mimics the phosphorylation of S315 in mmCx36, or a truncated Cx36 variant (**c**). Gap junctions were detected between adjacent cells and reconstructed (**d**). (**e**) Quantification of gap junction volume showed that the S315D mutation did not alter gap junction size in transfected HEK293 cells, in contrast to the truncation which led to significantly smaller gap junctions. Values are given as mean ± SD; n.s., not significant; **p < 0.01; Mann–Whitney test, n = 123/138 cell pairs from 4 transfections. Scale: 10 µm.

Earlier studies^23,24^ showed that phosphorylation of connexins may alter the internalization rate and affect gap junction size. Therefore, we tested in cultured cells whether the phosphorylation of S315 has an influence on the size of gap junctions. As we have successfully used mmCx36 in a mammalian expression system to measure gap junction volume^25^ and sequences for mouse and perch connexins are conserved with respect to the phosphorylated serine and PDZ binding domain (Fig. 1b), we cloned an mmCx36 variant, in which S315 was mutated to aspartate (S315D) to mimic the phosphorylation of the serine residue. HEK293 cells were transfected with either wild-type mmCx36 or the phosphomimetic construct and gap junctions were 3D reconstructed and measured in volume (Fig. 3) as described^25^. The volume of gap junctions between adjacent cells was similar between wild-type mmCx36 and the S315D mutant (Fig. 3a,b,e; mean ± SD cluster size in µm^3^: Cx36 WT: 7.4 ± 9.7; Cx36 S315D: 6.2 ± 5.5; *p* = 0.521, 123/138 cell pairs from 4 transfections, Mann–Whitney test). This suggests that phosphorylation of mmCx36 S315 does not affect gap junction size in HEK293 cells. However, when we compared gap junction volume between mmCx36 and a truncated mutant (Cx36 S318 ter), we found clusters to be significantly smaller in the truncated mutant (Fig. 3c–e, Cx36 WT: 7.4 ± 9.7; Cx36 S318 ter: 3.7 ± 3.2; *p* = 0.0011, 123/138 cell pairs from 4 transfections, Mann–Whitney test), consistent with an earlier report^20^.

### Does phosphorylation of S315 in mmCx36 change the interaction with MUPP1 in HEK293 cells?

**Figure 4:**
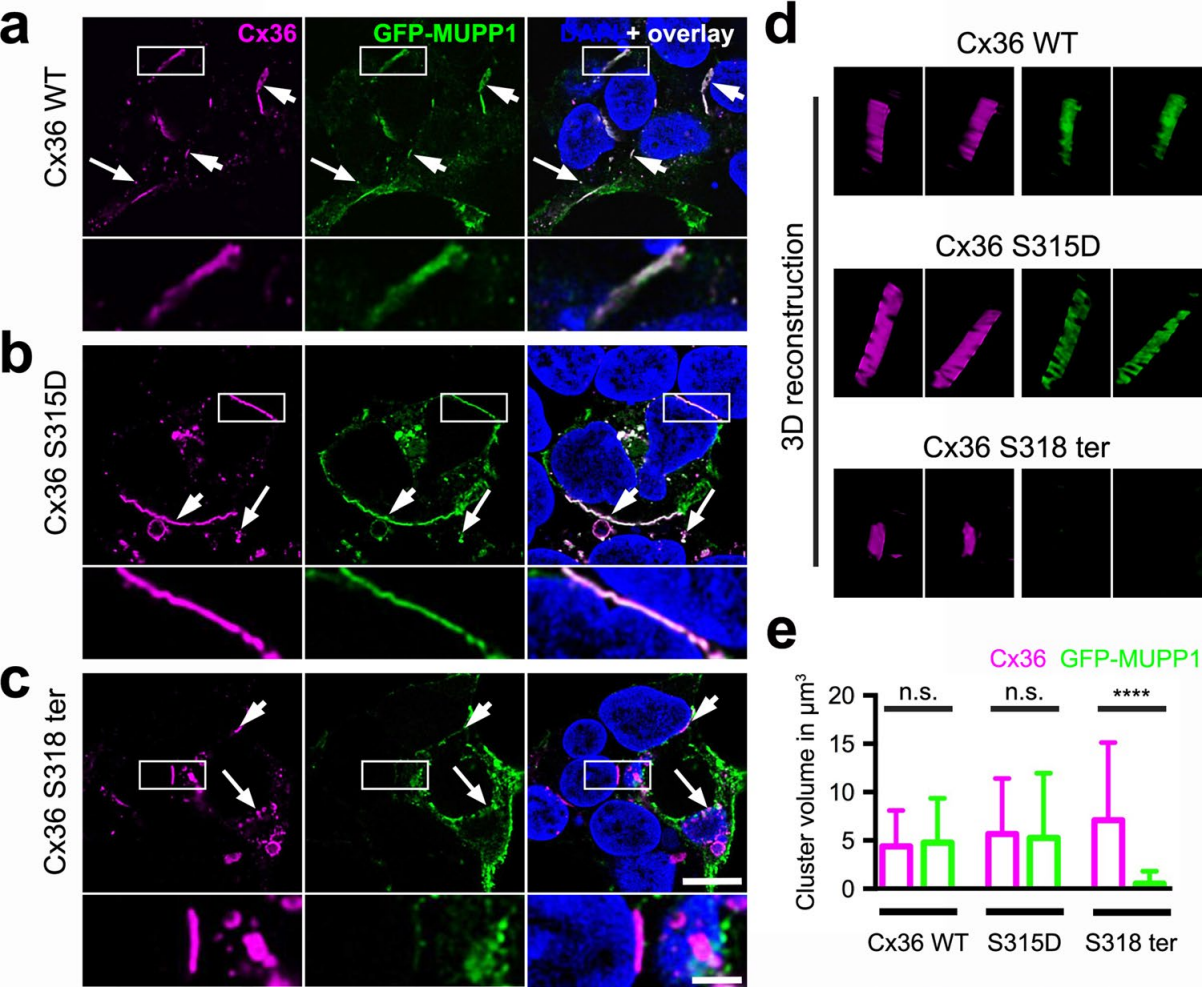
A phosphomimetic S315D mutation in mmCx36 did not alter the interaction with MUPP1-GFP in transfected HEK293 cells. (**a**–**c**) HEK293 cells co-transfected with GFP-MUPP1 and either mmCx36 WT (**a**), the phosphomimetic mutant Cx36 S315D (**b**), or a truncated mmCx36 variant (Cx36 S318 ter, **c**). Short arrows point to gap junction-like structures between two adjacent cells; long arrows indicate intracellular vesicles. Areas marked by the rectangles are shown in higher magnification in the lower panels. (**d**) 3D reconstructions of individual gap junctions. (**e**) Quantification of gap junction volume revealed that the phosphomimetic mutation of Cx36 (S315D) did not change the size of the gap junction and interaction with full length MUPP1. In contrast, truncation of mmCx36 at S318 (Cx36 S318 ter) abolished the interaction with MUPP1, which was consequently absent from gap junctions, while Cx36 S318 ter-containing gap junctions were still formed and normal in size. Values are given as mean ± SD; n.s., not significant; ****p < 0.0001, Mann–Whitney test, *n* = 40–44 cell pairs from 2 transfections. Scale: 10 µm, enlarged images: 5 µm.

Next, we asked whether phosphorylation of S315 alters the interaction of mmCx36 with MUPP1 (from rat, *Rattus norwegicus, rn*) in HEK293 cells. Again, we used the wild-type and phosphomimetic mutant (S315D) and cotransfected it with MUPP1, which was fused to GFP (GFP-MUPP1, Fig. 4). GFP-MUPP1 strongly colocalized with both constructs (Fig. 4a,b,d) but we did not find any differences between the volume of gap junctions for wild-type mmCx36 and the phosphomimetic mutant (mean ± SD cluster size in µm^3^: mm Cx36 WT: Cx36: 4.4 ± 3.7; MUPP1: 4.8 ± 4.6; *p* = 1; mmCx36 S315D: Cx36: 5.7 ± 5.7; MUPP1: 5.2 ± 6.7; *p* = 0.4; 34–45 cell pairs from 2 transfections, Mann Whitney test; Fig. 4e). Thus, mimicking the phosphorylation of mmCx36 at S315 did not affect the interaction with MUPP1.

As an earlier report^20^ showed that MUPP1 binds to the very C-terminal tail of Cx36, we also cotransfected GFP-MUPP1 with a truncated version of mmCx36. As expected, GFP-MUPP1 was not detected in Cx36-containing gap junctions (Fig. 4c–e) and consequently, the volume of clusters formed by GFP-MUPP1 and mmCx36 S318 ter differed significantly (mean ± SD cluster size in µm^3^: mmCx36 WT: Cx36: 4.4 ± 3.7; MUPP1: 4.8 ± 4.6; *p* = 1; S318 ter: Cx36: 7.1 ± 8.0; MUPP1: 0.5 ± 1.3; *p* < 0.0001; 31–45 cell pairs from 2 transfections, Mann Whitney test, Fig. 4e). This confirmed that binding of PDZ 10 of MUPP1 to mmCx36 is mediated by the PDZ binding domain at the very C-terminal end of the protein. Interestingly, while several studies^19,25^ earlier reported that the truncated protein (mmCx36 S318 ter) fails to form gap junctions in transfected HeLa cells, the same construct assembled into gap junctions in HEK293 cells, suggesting that the assembly machinery differs between cell lines.

### Does glutamate-induced activation of CaMKII change the interaction of mmCx36 and PSD95 in HeLa cells?

**Figure 5:**
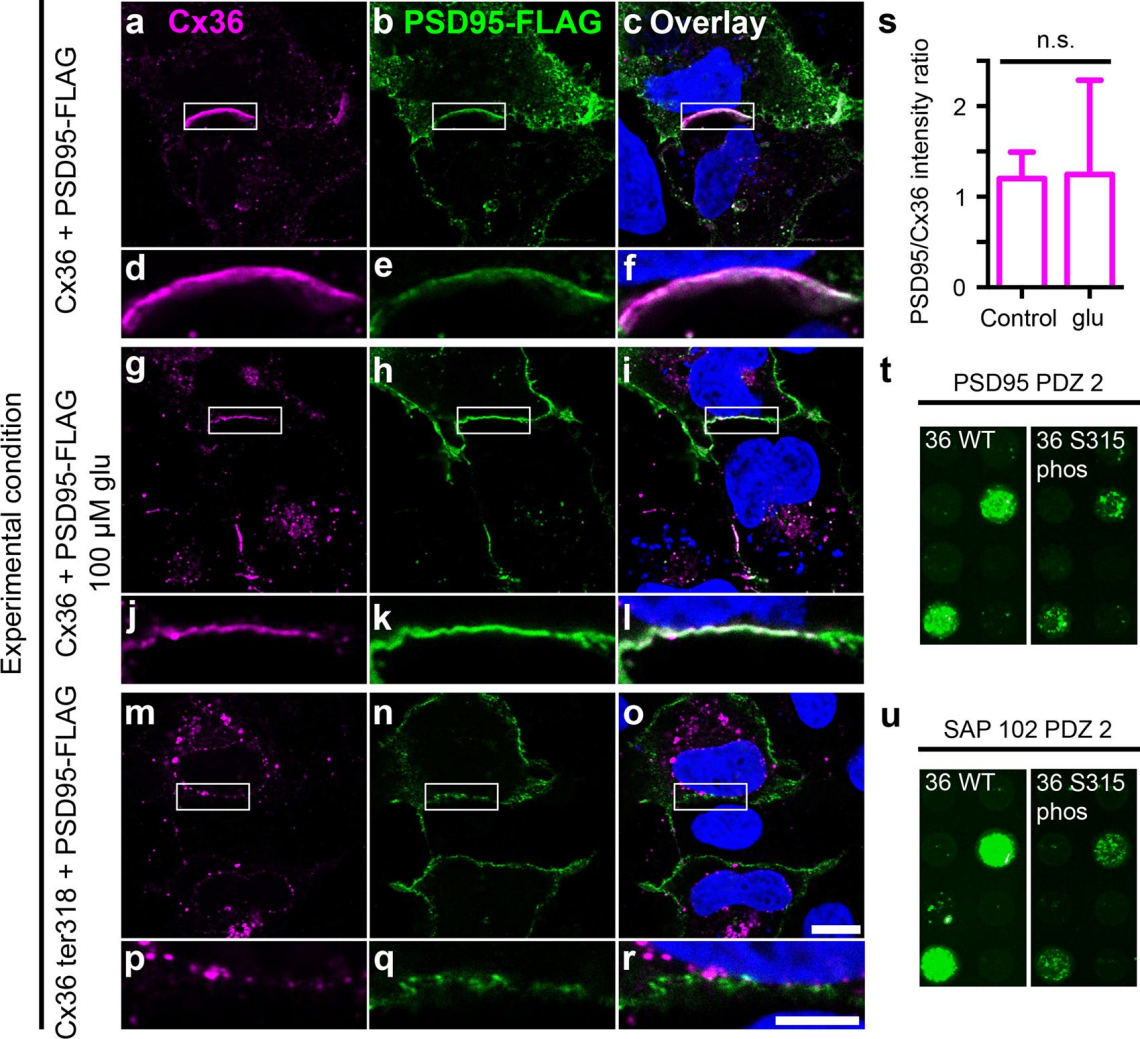
Activation of CaMKII does not cause any apparent changes in Cx36/PSD95 association. (**a**–**f**) Cx36 and PSD-95-FLAG colocalized at gap junctions in transfected HeLa cells. (**g**–**l**) Activation of CaMKII by treatment with glutamate (glu) did not affect the localization of PSD95 at gap junctions. (**m**–**r**) Expression of the Cx36 S318 ter mutant prevented PSD95 binding, indicating that this interaction requires a PDZ domain. Please note that hardly any gap junctions were formed and colocalization was absent also from intracellular vesicles, which showed colocalization when the PDZ domain of mmCx36 was still present (**a**–**l**). (**s**) Activation of CaMKII via glutamate (glu) did not affect the PSD95/Cx36 intensity ratio at gap junctions. 12 gap junctions in each condition were quantified. (**t, u**) Microarrays revealed phosphorylation-dependent reduction of mmCx36 C-terminal binding to PSD95 PDZ2 and SAP102 PDZ2. Scale: 10 µm.

Our results so far suggest that the phosphomimetic mutants might not be a good substitution for phosphorylated serine residues because they differ in charge and size (see “Discussion”). We next tried to circumvent this problem and tested the association of PSD95 and mmCx36 in HeLa cells upon activation of CaMKII. PSD95 was chosen because it closely associates with Cx36-containing gap junctions in photoreceptors^26^. To stimulate endogenous CaMKII activity and consequently phosphorylation of mmCx36, we treated transfected HeLa cells with 100 µM glutamate for 15 min (Fig. 5a–l) as we recently showed that this treatment increases gap junction coupling approximately two-fold via the activation of endogenous NMDA receptors and CaMKII^27^. However, when we compared the intensity ratio of PSD95 and Cx36 at gap junctions between adjacent HeLa cells, we did not detect any significant differences between untreated (control, Fig. 5a–f,s) and treated HeLa cells (Fig. 5g–l,s; p = 0.889, two-tailed t-test; n = 12 gap junctions per condition).

We also tested binding of mmCx36 and PSD95 (PDZ domain 2) in a microarray. SAP102 was also added due to its similarity to PSD95, sharing with it three PDZ domains, an SH3 domain and a C-terminal guanylate kinase domain. For both proteins, phosphorylation of S315 in mmCx36 led to a decrease in binding of the PDZ domain (Fig. 5t,u; Supplementary Fig. 1). However, even when S315 was phosphorylated, PDZ domain binding was not abolished but only reduced. The fact that we see similar results with two closely related proteins (PSD95 and SAP102) corroborates the hypothesis of a universal phosphorylation-dependent switch. Additionally, the rather moderate effect on the binding affinity could explain the preserved association of PSD95 and Cx36 we see in transfected HeLa cells stimulated with glutamate.

To confirm that the association of mmCx36 and PSD95 is mediated via the PDZ binding domain in the C-terminal tail of Cx36, we transfected HeLa cells with the truncated Cx36 protein (mmCx36 S318 ter) and FLAG-tagged PSD95. As expected, PSD95 showed virtually no colocalization with Cx36 (Fig. 5m–r).

In summary, in vitro experiments using C-terminal peptides of mmCx36/maCx35, which carried a phosphorylation at S315/298, showed that phosphorylation of a serine residue close to the C-terminal PDZ binding site of Cx36/35 may change the interaction with different protein partners, such as MUPP1 and 14–3–3 γ/η. However, cell culture experiments with mmCx36 constructs carrying a phosphomimetic mutation (S315D) failed to reproduce this result, presumably because the aspartate residue is not a good substitution for a phosphorylated serine in this case. Also, activation of CaMKII via endogenously expressed glutamate receptors did not lead to detectable differences in the association of mmCx36 and its scaffolds.

## Discussion

Here, we provide evidence that phosphorylation of a serine residue in the C-terminal tail of Cx36/Cx35 may serve as a molecular switch to regulate the binding of different scaffolding proteins to Cx36/35. This concept connects two well-known mechanisms that were shown to regulate Cx36-containing gap junctions: phosphorylation by CaMKII^9,11^ and the PDZ-dependent association with scaffolding proteins^20^. As CaMKII-mediated phosphorylation was shown to be linked to NMDA receptor activation^9,11^, this switch may provide a substrate for activity-driven plasticity at Cx36-containing electrical synapses.

Interestingly, the phosphorylation-induced switch seems to act in two different directions: it increases binding to 14–3–3 proteins and decreases binding to PDZ 10 of MUPP1. Most likely, two different mechanisms are at work: (1) the binding to MUPP1 is likely mediated via the PDZ binding site at the very C-terminal end of Cx36/35 and this binding affinity changes when S315/298 is phosphorylated. (2) The binding to 14–3–3 proteins may be direct, via the phosphorylated S315/298, as was reported for Cx43^28^. However, the Cx36/35 sequence does not contain a classical consensus 14–3–3 binding site^29^. Still, earlier studies reported a similar switching mechanism in viral^30,31^ but also mammalian proteins^32^, such as the receptor tyrosine kinase ERBB4 and the inward rectifier potassium channel IRK1 (Kir2.1): phosphorylation of a PDZ binding motif decreases PDZ binding activity and mediates interaction with 14–3–3 proteins^31^. This suggests that this switch is a rather general means to regulate protein–protein interactions.

Our results are based on a protein microarray approach, which resulted in several candidate proteins potentially affected by Cx36/35 S315/298 phosphorylation. Pull-down experiments were used to test some of the candidate proteins for direct interaction which confirmed the binding interactions, but only confirmed the phosphorylation-dependent switch for MUPP1 and 14–3–3 proteins (γ and η isoforms).

Co-expression studies in HEK293 cells were employed to test whether the phosphorylation-dependent interaction with MUPP1 affects the size of mmCx36-containing gap junctions. This was not the case; introduction of a phosphomimetic mutation (S315D) did not affect the volume of Cx36-containing gap junctions in HEK293 cells. This was somewhat surprising because introduction of a phosphomimetic mutation at Cx43 S373 affects gap junction formation^24^. However, there may be several explanations for the absence of an effect on gap junction size: First, the phosphomimetic serine-to-aspartate mutation—although also introducing a negative charge, just like phosphorylation—might not be similar enough to the real phosphorylation because phosphate groups contain an additional negatively charged oxygen atom and differ in their ionic shell from negatively charged amino acid residues^33^. Thus, phosphate groups create a different chemical landscape^33,34^ and may be more potent in disrupting protein–protein interactions. In line with this, an earlier study reported that also 14–3–3 proteins do not bind to phosphomimetic threonine-to-aspartate mutations^30^. Lack of efficacy for the phosphomimetic mutation may also explain why binding of MUPP1 to the PDZ binding domain of Cx36 was not affected in the S315D mutants in HEK293 cells, whereas it was when maCx35 peptides were used that carried a phosphorylation at S298 (Cx35 S298 phos).

Second, phosphorylation of S315/298 may only moderately affect PDZ binding affinity: our microarray data revealed some residual binding of MUPP1-PDZ-10 to S298 phos, which might also explain the persistent colocalization between MUPP1 and the phosphomimetic mmCx36 mutant, which we observed in the cell culture system. We obtained similar results for PDZ2 of PSD95 and SAP102: association clearly depended on the PDZ binding domain in the C-terminal tail but phosphorylation only decreased the binding and did not entirely prevent it.

Finally, phosphorylation of S315 may not affect gap junction size but channel gating^17^, by influencing the orientation of intracellular domains in the Cx36 protein. Here, the interaction with 14–3–3 proteins may come into play, which we found to be stronger when S298 was phosphorylated in Cx35. 14–3–3 proteins are able to bind two phosphorylated residues simultaneously^35^. Thus, it is tempting to speculate that 14–3–3 proteins might simultaneously interact with two phosphorylated amino acids within different domains of Cx36/35, forming an intramolecular bridge and thereby affecting Cx36/35 gating.

Our attempts to use endogenous phosphorylation of S315 also failed to demonstrate a detectable phosphorylation-driven switch in association of Cx36 with PDZ scaffolds (Fig. 5a–l). Even when we increased the amount of CaMKII by overexpressing it, we did not detect any apparent changes in the colocalization of Cx36 and PSD95 (Supplementary Fig. 4). It is important to note that while glutamate stimulation increases CaMKII phosphorylation of Cx36, and S315 is an optimal CaMKII phosphorylation site, we do not know how complete phosphorylation is in vivo. Work from Bukauskas et al.^36^ showed that less than 5% of all connexons present in a gap junction plaque contribute to gap junction conductance, and only this small fraction of active channels needs to be phosphorylated to enhance coupling. Thus, it seems likely that confocal microscopy is not sensitive enough to detect these subtle changes. Furthermore, a change in the binding affinity of a small fraction of gap junction channels for PDZ scaffolds may free those channels to engage in conformational changes, including those potentially induced by 14–3–3 protein binding, that regulate coupling, while maintaining the important associations of the PDZ scaffolds with the plaque as a whole.

Phosphorylation in gap junction proteins is very common and serves different functions. Here, we provide evidence for a molecular mechanism that allows the Cx36/35 protein to recruit different interaction partners to the synapse in response to activity-related calcium entry and subsequent activation of CaMKII. This again confirms the notion that electrical synapses share many properties with their chemical counterparts when it comes to synaptic plasticity.

## Methods

### Protein microarray

Purified GST fusion proteins (listed in Fig. 1c and Supplementary Fig. 1) were spotted onto nitrocellulose-coated glass slides with an Aushon 2470 microarray robot; each protein was spotted in duplicate. Peptides corresponding to the C-terminus tip of maCx35 and mmCx36, their phosphorylated forms, a C-terminally truncated form of maCx35 and a scrambled form of mmCx36, each with an N-terminal biotin, were synthesized by Genscript. 10 µg of peptide was pre-bound to 5 µg Cy3-streptavidin (GE Health Sciences) in 500 µl phosphate buffered saline with 0.1% Tween-20 (PBST) and unbound streptavidin was removed by clearing with biotin-agarose beads (Sigma). Array slides were probed with the labeled peptide probe at 4 °C overnight. Slides were washed with PBST and scanned with a GenePix 4200A scanner (Molecular Devices).

### Protein purification

Recombinant proteins/protein domains (Table 1) were expressed in BL21 cells. Expression was induced with 1 mM isopropyl-β-d-thiogalactopyranoside (IPTG). After expression, cells were lysed with lysozyme (1 mg/ml) and insoluble material was separated by centrifugation at 17,000 rpm for 40 min. The supernatant was applied to a glutathione sepharose column and incubated overnight on a rotating platform at 4 °C. Non-bound material was removed at the next day and the column was washed several times with binding buffer (containing 150 mM NaCl, 50 mM Tris, pH 7.4, and 10 mM dithiothreitol). Proteins were eluted with binding buffer, supplemented with 10 mM reduced glutathione. For purification of His-tagged proteins, supernatants were applied to a nickel column. After flow-through of non-bound material, the column was washed with washing buffer (500 mM NaCl, 20 mM imidazole and 20 mM Tris, pH 7.5–6). Captured proteins were eluted with elution buffer (150 mM NaCl, 300 mM imidazole and 20 mM Tris, pH 7.5–6).

### Peptide pull-downs

For peptide pull-downs (n = 2–4), 100 µl of µMACS streptavidin beads (Miltenyi Biotec) were mixed with 0.25 µg of maCx35 peptides and equal amounts of purified PDZ domains/proteins for each condition in phosphate buffered saline (PBS) with 0.05% P20 (P20 is 20% Tween). The samples were incubated on ice for an hour and applied to a magnetic column for isolation. After several washes, adsorbed proteins were eluted with pre-heated (95 °C) elution buffer, containing 50 mM Tris HCl (pH 6.8), 50 mM dithiothreitol, 1% SDS, 1 mM EDTA, 0.005% bromophenol blue, 10% glycerol. SDS-PAGE (10% gels) and western blot analysis were performed as previously described^25^.

### Constructs and HEK293/HeLa cell transfections

Three different mmCx36 constructs (1. full-length mmCx36, 2. mmCx36 S315D, 3. mmCx36 S318 ter) were cloned into the pRK5 vector (BD Pharmingen, San Diego, CA, USA). The PDZ domain 10 of rnMUPP1 was cloned into the PetM11 vector. All constructs were sequenced for accuracy. PSD95-FLAG in pcDNA5/FRT/TO was a gift from Dr. Wei-dong Yao (Addgene plasmid #15463; RRID:Addgene_15463). This clone was initially generated by Zhang et al.^37^. Monomeric eGFP-CaMKIIa fusion construct was a gift of Dr. M. Neal Waxham (University of Texas Health Science Center at Houston)^38^. HEK293 and HeLa cells were plated at a density of 5 × 10^5^ cells in a Petri dish (6 cm diameter), including 12 mm coverslips, in 5 ml Dulbecco’s Modified Eagle Medium. Cells were transfected with 0.6–1.4 µg/ml DNA using Lipofectamine (Life Technologies), 24 h after seeding. All transfections were done as triplicates and independently performed at least twice.

To stimulate endogenous CaMKII activity, transfected HeLa cells were treated with serum-free media containing 1 mM glycine and 100 µM glutamate for 15 min in a cell culture incubator (37 °C, 5% CO_2_). Afterwards, HeLa cells were fixed in 2% PFA in PBS for 15 min and prepared for confocal scans.

### Immunocytochemistry

HEK293 and HeLa cells were fixed with 2% paraformaldehyde 48 h after transfection. Cells were washed with 0.1 M phosphate buffer (PB, pH 7.4) and incubated in primary antibodies (Table 2) in PB at 4 °C overnight. After washing with PB, secondary antibodies conjugated to Alexa 488 and Alexa 568 (1:500, Thermo Fisher Scientific) were applied for 2 h at room temperature. Antibodies were diluted in PB containing 10% normal goat serum and 0.5% Triton-X100. After extensive washing, coverslips were mounted in Vectashield with DAPI (Vector Laboratories) and sealed with nail polish.

**Table 2:** Primary antibodies used in this study.

Antibody	Host, type	Dilution	Source cat. (no.)
Cx36, clone: 1E5H5	Mouse, monoclonal	1:500	Thermo Fisher Scientific, 37-4600
Cx36	Rabbit, polyclonal	1:500	Thermo Fisher Scientific, 36-4600
Cx36	Goat, polyclonal	1:500	Santa Cruz, sc-14904
FLAG	Rabbit, polyclonal	1:500	Thermo Fisher Scientific, PA1-984B
GFP	Chicken, polyclonal	1:500	Sigma Aldrich, AB16901
GST	Goat, polyclonal	1:5000	GE Healthcare, 27-4577-01V
RGS·His	Mouse, monoclonal	1:500	Qiagen, 34610

### Confocal microscopy and image analysis

The volume of Cx36-containing gap junctions between adjacent HEK293 cells was quantified as described^25^. Quantification of gap junction volume was performed blindly, i.e., persons imaging the gap junctions and determining the volume did not know the respective transfection condition as it was done by somebody else. Confocal stacks were acquired using a Leica SP8 confocal microscope equipped with a 63 × HCX PL APO oil immersion objective (NA 1.4). Stacks were deconvolved with Huygens Essential deconvolution software (using theoretical point spread functions) and further processed in Fiji (https://fiji.sc/, 2020^39^). After background subtraction and histogram normalization, stacks were thresholded using the automated Otsu threshold. Gap junction clusters were detected with the *3D Simple Segmentation* plugin and their volume was measured using the *3D Manager* plugin in Fiji. Data were tested for statistical differences using the Mann–Whitney test in Prism 6 (GraphPad Software), at an alpha level of 0.05.

The intensity ratio of mmCx36 and PSD95 located at gap junctions was calculated using the *measure* function in Fiji. A linear region of interest (2 µm in length) was placed in a gap junction and the average intensity along this ROI was measured for each channel. The average intensity ratio of both channels in each experimental condition was used as a measure of PSD95/Cx36 association.

Images of co-transfected cells are shown as single scans. Single transfected cells are presented as a maximum intensity projections of 12 sections (z-distance: 0.2 µm). Contrast and brightness were adjusted for presentation purposes in Fiji.

## Supplementary information

Supplementary Information

## Data Availability

The datasets generated during and/or analysed during the current study are available from the corresponding author on reasonable request.
