# Supplementary material for: Phosphorylation of Connexin36 near the C-terminus switches binding affinities for PDZ-domain and 14–3–3 proteins in vitro
Source: Sci Rep. 2020 Oct 27;10:18378. doi: 10.1038/s41598-020-75375-0 (PMC7592057; doi:10.1038/s41598-020-75375-0)
Supplement: Supplementary file 1 — Supplementary Information [file 41598_2020_75375_MOESM1_ESM.pdf]

Phosphorylation of Connexin36 near the C-terminus switches binding affinities for PDZ-domain and 14-3-3 proteins *in vitro*

Stephan Tetenborg[1,2], Helen Y. Wang[2], Lena Nemitz[3], Anne Depping[1], Alexsandra B. Espejo[4], Jaya Aseervatham[2], Mark T. Bedford[4], Ulrike Janssen-Bienhold[3,5], John O'Brien[2,#], Karin Dedek[1,5,#,*]

1 Animal Navigation/Neurosensorics, Institute for Biology and Environmental Sciences, University of Oldenburg, Oldenburg, Germany
2 Ruiz Department of Ophthalmology & Visual Science, The University of Texas Health Science Center at Houston, Houston, TX 77030, USA
3 Visual Neuroscience, Dept. of Neuroscience, University of Oldenburg, Oldenburg, Germany
4 Department of Epigenetics and Molecular Carcinogenesis, University of Texas M.D. Anderson Cancer Center, Smithville, TX 78957, USA
5 Research Center Neurosensory Science, University of Oldenburg, Oldenburg, Germany
# These authors contributed equally.

C-Terminal Domain Readers Array

PDZ	PDZ	PDZ	14-3-3 / 14-3-3 like
A 1) α-1-syntrophin (1/1)/Q61234	B 1) Cipp (3/10)/Q63ZW7	C 1) Harmonin (2/3)/Q9ES64	D 1) sigma/NP_006133
A 2) β1-syntrophin (1/1)/Q99L88	B 2) Cipp (5/10)/Q63ZW7	C 2) HtrA1 (1/1)/Q9QZK6	D 2) beta/alpha/NP_647539.1
A 3) γ1-syntrophin (1/1)/Q925E1	B 3) Cipp (8/10)/Q63ZW7	C 3) HtrA3 (1/1)/Q9D236	D 3) epsilon/NP_006752
A 4) γ2-syntrophin (1/1) /Q925E0	B 4) Cipp (9/10)/Q63ZW7	C 4) Interleukin 16 (1/4)/Q9QZP6	D 4) gamma/CAG46702
A 5) Chapsyn-110 (2/3)/Q91XM9	B 5) Cipp (10/10)/Q63ZW7	C 5) LARG (1/1)/Q8R4H2	D 5) eta/CAG30498
A 6) Chapsyn-110 (3/3)/Q91XM9	B 6) Radil (1/1)/Q69Z89	C 6) LIN-7A (1/1)/Q8JZS0	D 6) theta/NP_006817
A 7) Dlgh3 (1/1)/Q6XE40	B 7) Erbin (1/1)/Q80TH2	C 7) Lin7c (1/1)/O88952	D 7) zeta/delta/NP_663723
A 8) Dvl1 (1/1)/Q60838	B 8) GRASP55 (1/1)/Q99JX3	C 8) Lnx1 (2/4)/O70263	D 8) SMG5/NP_056142
A 9) Dvl2 (1/1)	B 9) Grip1 (6/7)/Q925T6	C 9) Lnx1 (3/4)/O70263	D 9) SMG7/NP_963862
A 10) Dvl3 (1/1)	B 10) Grip2 (5/7)/E0CXS4	C 10) Lrrc7 (1/1)/Q80TE7	

PDZ	PDZ	PDZ
E 1) Magi-1 (2/6)/Q6RHR9	F 1) Mpp7 (1/1)/Q8BVD5	G 1) NHERF-1 (1/2)/P70441
E 2) Magi-1 (4/6)/Q6RHR9	F 2) MUPP1 (5/13)/Q8VBX6	G 2) NHERF-1 (2/2)/P70441
E 3) Magi-1 (6/6)/Q6RHR9	F 3) MUPP1 (10/13)/Q8VBX6	G 3) NHERF-1 FL/P70441 *
E 4) Magi-2 (2/6)/Q9WVQ1	F 4) MUPP1 (11/13)/Q8VBX6	G 4) NHERF-2 (1/2)/Q9JHL1
E 5) Magi-2 (5/6)/Q9WVQ1	F 5) MUPP1 (12/13)/Q8VBX6	G 5) NHERF-2 (2/2)/Q9JHL1
E 6) Magi-2 (6/6)/Q9WVQ1	F 6) MUPP1 (13/13)/Q8VBX6	G 6) NHERF-2 FL/Q9JHL1 *
E 7) Magi-3 (5/6)/Q9EQJ9	F 7) nNOS (1/1)/Q9Z0J4	G 7) Pdzk1 (1/4)/NHERF-3/Q9JIL4
E 8) Mals2 (1/1)/lin-7/Q9HAP6	F 8) OMP25 (1/1)/Q8K4F3	G 8) Pdzk1 (3/4)/NHERF-3/Q9JIL4
E 9) Magi-3 (1/6)/Q9EQJ9	F 9) PAR-3 (3/3)/Q99NH2	G 9) Pdzk3 (1/4)/NHERF-4/Q99MJ6
E 10) Semcap3 (1/2)/Q69ZS0	F 10) Shank3 (1/1)/Q4ACU6	G 10) Pdzk3 (3/4)/NHERF-4/Q99MJ6

PDZ	PDZ	PDZ
H 1) PAR6B (1/1)/Q9JK83	I 1) SAP102 (2/3)/P70175	J 1) Shroom (1/1)/Q9QXN0
H 2) Pdlim5 (1/1)/Q3UGD0	I 2) SAP102 (3/3)/P70175	J 2) SLIM (1/1)/Q8R1G6
H 3) Pdzk11 (1/1)/Q9CZG9	I 3) SAP97 (1/3)/Q811D0	J 3) Tiam2 (1/1)/Q6ZPF3
H 4) PDZ-RGS3 (1/1)/Q9DC04	I 4) SAP97 (2/3)/Q811D0	J 4) Whirlin (3/3)/Q5MLF8
H 5) PSD95 (1/3)/Q62108	I 5) SAP97 (3/3)/Q811D0	J 5) ZO-1 (1/3)/P39447
H 6) PSD95 (2/3)/Q62108	I 6) Scrb1 (3/4)/Q80U72	J 6) ZO-1 (2/3)/P39447
H 7) PSD95 (3/3)/Q62108	I 7) Shank1 (1/1)/D3YZU1	J 7) ZO-2 (1/3)/Q9Z0U1
H 8) PTP-BL (2/5)/Q64512	I 8) Pdzk3 (1/6)/E9Q1M1	J 8) ZO-3 (1/3)/Q9QXY1
H 9) PAR3B (1/3)/Q5SV53	I 9) Pdzk3 (2/6)/E9Q1M1	J 9) Scrb1 (1/4)/Q80U72
H 10) TIP-1 (1/1)/Q9DBG9		J 10) Scrb1 (2/4)/Q80U72

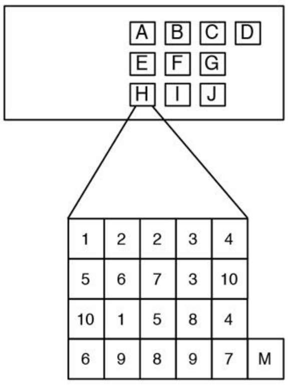

*= Non-Codon Optimized Construct

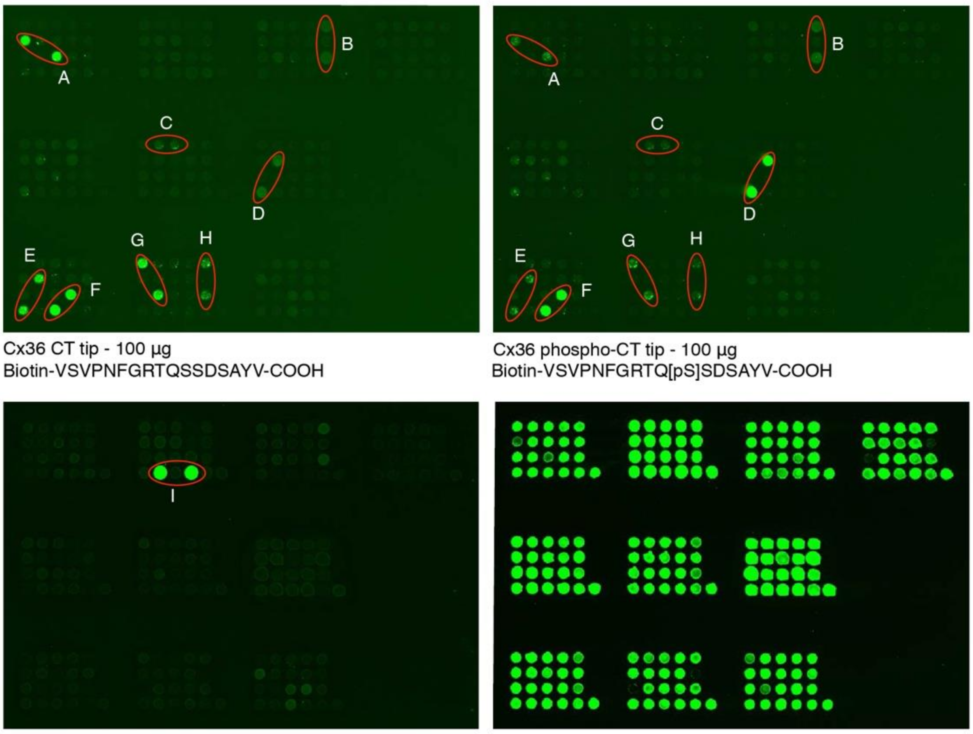

Cx36 CT tip - 100 µg
Biotin-VSVPNFGRTQSSDSAYV-COOH

Cx36 phospho-CT tip - 100 µg
Biotin-VSVPNFGRTQ[pS]SDSAYV-COOH

Scrambled - 927206 - 100 µg
Biotin-QRTDNKSRALSGYRVEV-COOH

αGST

A. Chapsyn-110 (2/3) PDZ
B. Interleukin 16 (1/4) PDZ
C. MUPP1 (5/13) PDZ
D. NHERF-2 FL
E. PSD95 (2/3) PDZ
F. PTP-BL (2/5) PDZ
G. SAP102 (2/3) PDZ
H. SAP97 (2/3) PDZ

I. Grip1 (6/7) PDZ

Supplementary Figure 1. C-terminal domain readers microarray probed with mmCx36 peptides. Note that although the scrambled peptide sequence was generated by reassigning residues of Cx36 peptides with a random number generator, a PDZ-binding site was generated that binds to Grip1.

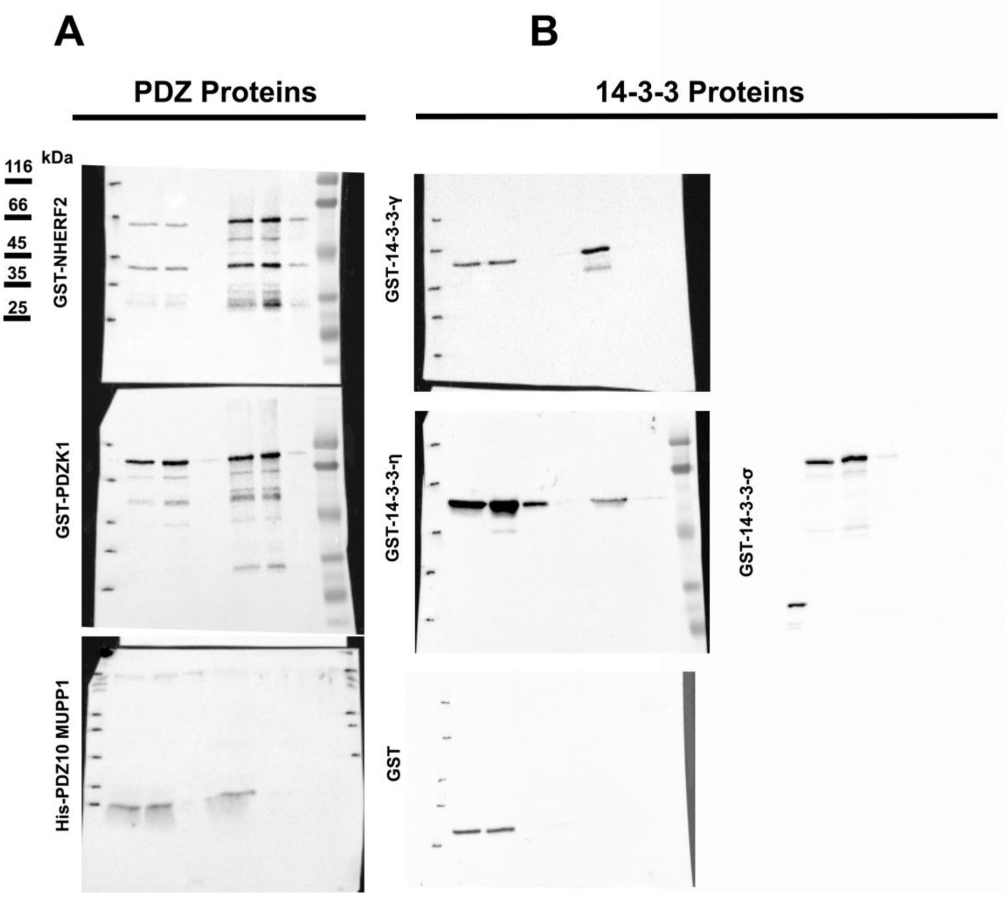

Supplementary Figure 2. Original blots of pull-down experiments. **(A)** Original blots of pull-down experiments with PDZ proteins. **(B)** Original blots of pull-down experiments with GST-tagged 14-3-3 proteins.

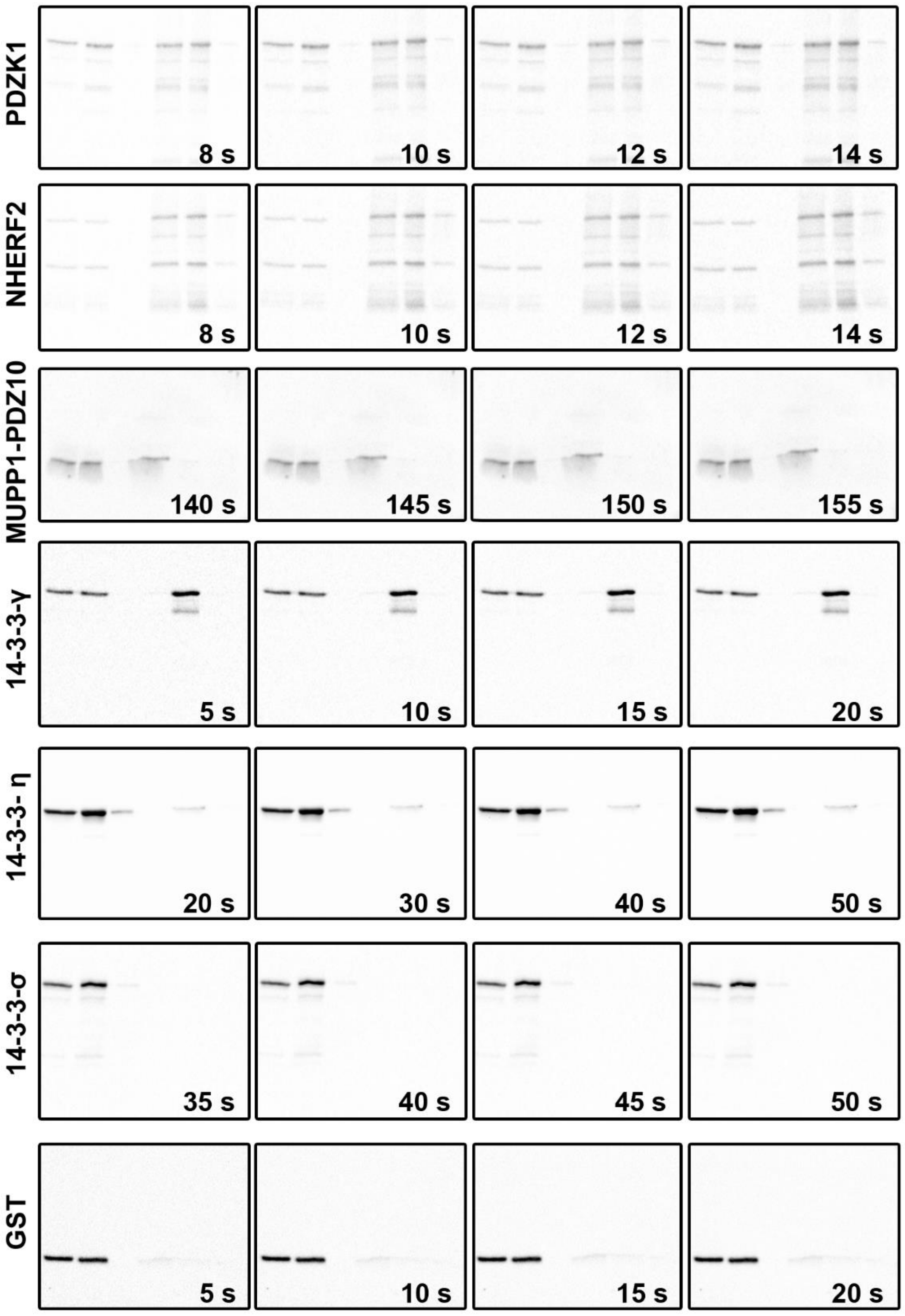

Supplementary Figure 3. Blots of pull-down experiments with variable exposure times.

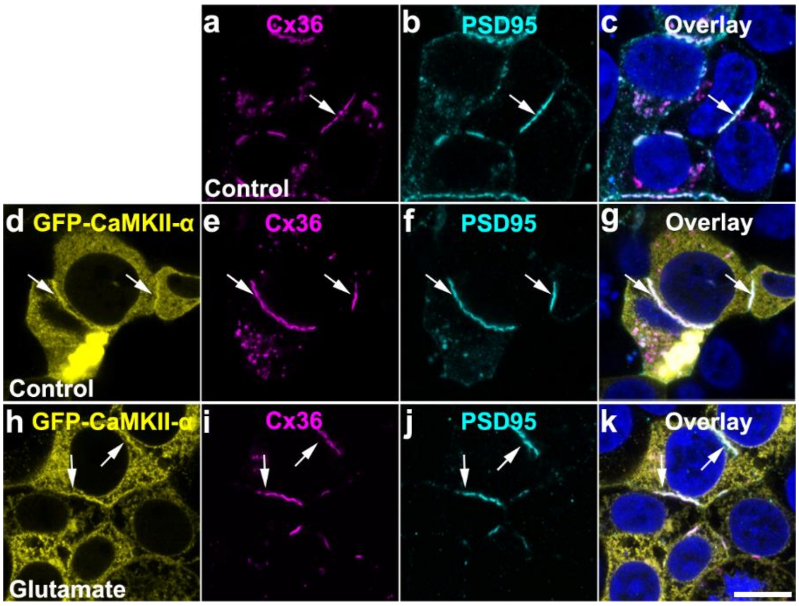

Supplementary Figure 4. Activation of co-expressed CaMKII did not cause any apparent changes in Cx36/PSD95 association. (a-c) Colocalization of Cx36 and PSD95 at gap junctions (arrow) in co-transfected HEK293 cells. **(d-g)** Co-expression of CaMKII-α with Cx36 and PSD95 in HEK293 cells. **(h-k)** Activation of co-transfected CaMKII by treatment with glutamate (100 µM) did not affect the localization of PSD95 at Cx36-containing gap junctions. Arrows in a-k point to gap junctions between two adjacent cells. Scale: 10 µm.
